# A Novel Approach to Automatically Balance Flow in Profile Extrusion Dies Through Computational Modeling

**DOI:** 10.3390/polym17111498

**Published:** 2025-05-28

**Authors:** Gabriel Wagner, João Vidal, Pierre Barbat, Jean-Marc Gonnet, João M. Nóbrega

**Affiliations:** 1 Institute for Polymers and Composites—IPC, University of Minho, 4800-058 Guimarães, Portugal; jpovidal@gmail.com; 2 Michelin Ladoux Campus RDI, 63118 Cébazat, France; pierre.barbat1@michelin.com (P.B.); jean-marc.gonnet@michelin.com (J.-M.G.)

**Keywords:** tire manufacturing, extrusion die optimization, Bayesian optimization, OpenFOAM, computational fluid dynamics, flow balancing

## Abstract

This work presents a novel fully automated computational framework for optimizing profile extrusion dies, aiming to achieve balanced flow at the die flow channel outlet while minimizing total pressure drop. The framework integrates non-isothermal, non-Newtonian flow modeling in OpenFOAM with a geometry parameterization routine in FreeCAD and a Bayesian optimization algorithm from Scikit-Optimize. A custom solver was developed to account for temperature-dependent viscosity using the Bird–Carreau–Arrhenius model, incorporating viscous dissipation and a novel boundary condition to replicate the thermal regulation used in the experimental process. For optimization, the die flow channel outlet cross-section is discretized into elemental sections, enabling localized flow analysis and establishing a convergence criterion based on the total objective function value. A case study on a tire tread die demonstrates the framework’s ability to iteratively refine internal geometry by adjusting key design parameters, resulting in significant improvements in outlet velocity uniformity and reduced pressure drop. Within the searching space, the results showed an optimal objective function of 0.2001 for the best configuration, compared to 0.7333 for the worst configuration, representing an enhancement of 72.7%. The results validate the effectiveness of the proposed framework in navigating complex design spaces with minimal manual input, offering a robust and generalizable approach to extrusion die optimization. This methodology enhances process efficiency, reduces development time, and improves final product quality, particularly for complex and asymmetric die geometries commonly found in the automotive and tire manufacturing industries.

## 1. Introduction

The profile extrusion of thermoplastic materials plays a crucial role in the manufacturing of various products with complex cross-sectional geometries, including window frames, automotive trims, and sealing systems [1,2]. The extrusion die, which shapes the molten polymer into the final profile geometry, is a key component of this process. Its design significantly influences the dimensional accuracy, appearance of the extrudate, production efficiency, and overall product quality [1,3,4,5].

The design of profile extrusion dies presents notable challenges due to the interaction of several factors [6,7], ranging from processing conditions to post-extrusion phenomena like swelling effect and shrinkage due to cooling, with flow balance being the most critical [1]. Uneven flow at the die outlet can lead to geometric distortions after the polymer melt exits the extrusion die flow channel or during cooling and solidification, impeding the ability to produce a specific profile and resulting in dimensional deviations and defects such as warping, extrudate swell, and surface irregularities that adversely affect the quality of the final product [8,9,10]. Additionally, post-extrusion phenomena—such as thermal contraction, residual stress development, and variations in cooling rate—complicate the process further, making uniform flow at the die exit essential for ensuring profile fidelity and product performance [1].

Consequently, achieving balanced flow distribution within the die is a fundamental requirement, ensuring that all sections of the profile exhibit a uniform flow distribution, thereby minimizing post-extrusion distortions [11]. However, attaining this balance is complicated by the complex geometries often required, particularly in industries such as automotive and tire manufacturing, where asymmetrical and varying thickness profiles are common.

Numerical modeling has emerged as a powerful tool to support extrusion die design [12] by enabling accurate simulation of the non-isothermal, non-Newtonian flow of polymer melts through intricate flow channels [13,14,15]. Such simulations allow designers to evaluate and optimize flow distribution prior to manufacturing, thereby reducing reliance on costly trial-and-error iterations [16,17]. In this context, modeling plays a critical role in accelerating development, minimizing material waste, and enhancing precision [16].

Over recent decades, various approaches have been proposed to automate die design and achieve balanced flow distribution. These approaches include inverse design methods [13,18,19,20], parameterized optimization schemes [4,10,21,22,23,24,25], and surrogate-based methods [8,9]. While these strategies have demonstrated success in specific applications, they often possess inherent limitations due to the need to link geometric variables to velocity targets in particular die flow channel sections [11,26].

Parameterization methods that directly correlate geometric variables with local flow targets—such as those that segment the die outlet into elemental sections—offer computational efficiency but are typically confined to problems with relatively simple mappings between geometry and flow [11,20,21,25,27]. This limitation restricts their applicability to more complex profiles, such as those encountered in tire manufacturing, where interdependent effects across sections are prevalent and difficult to isolate [28]. Conversely, evolutionary algorithms can provide a more robust approach due to their efficacy in addressing nonlinear and multimodal problems. However, their computational demands can be prohibitive, making them impractical for large design spaces or resource-constrained environments [29]. In contrast, Bayesian optimization has recently emerged as a promising alternative, offering an efficient, data-driven strategy for global optimization in expensive black-box problems [20,30]. By constructing and iteratively updating a probabilistic surrogate model, Bayesian optimization facilitates intelligent sampling of the design space, significantly reducing the number of required simulations while maintaining global search capabilities [31]. This makes it particularly well-suited for high-fidelity, simulation-based extrusion die design, where evaluating each trial geometry can be computationally expensive.

This work proposes a new computational framework for the automated design of thermoplastic profile extrusion dies. This approach leverages Bayesian optimization to minimize flow imbalance at the die exit, integrating simulation tools implemented in OpenFOAM v2406 [32] with a geometry parameterization pipeline developed in FreeCAD 1.0 [33]. The optimization loop is managed using the Scikit-Optimize library [34], facilitating efficient exploration of the design space with minimal computational cost. By incorporating accurate non-isothermal flow modeling, open-source CAD generation, and adaptive optimization, the framework can accommodate a wide variety of profiles—ranging from symmetrical to highly asymmetric and simple to complex—without the limitations associated with existing parameter-linking or demanding evolutionary methods.

The remainder of the paper is structured as follows: Section 2 details the computational methodology, including solver development, integration with optimization routines, and system architecture. Section 3 describes the performed case study, including geometry, material properties, computational mesh, boundary conditions, and overall case setup. Section 4 presents the results and discussion, demonstrating the framework’s performance in optimizing a complex profile geometry. Finally, Section 5 concludes the paper by summarizing the main findings and outlining directions for future work.

## 2. Materials and Methods

### 2.1. The Modeling Solver

For numerical simulation, a customized flow solver, *viscousSimpleFoam*, was developed within the OpenFOAM framework. This solver enhances the standard steady-state incompressible flow solver *simpleFoam* [35] by integrating the energy conservation equation (Equation (1)) to allow prediction of the temperature distribution and account for temperature effects. The implementation ensures the simulation of fluid flow and heat transfer, with a shear-rate-dependent viscosity, enabling an accurate representation of the extrusion process. The energy conservation equation for steady-state incompressible fluid flow is given as follows [3]:

(1) $$\nabla (\rho c_{p}uT)-\nabla (\alpha \nabla T)=\tau :\nabla u,$$

where ρ is the density, $c_{p}$ the specific heat, u the velocity vector, T the temperature, α the thermal diffusivity, and τ the deviatoric stress tensor. The term τ:∇T represents the viscous dissipation, accounting for self-heating caused by internal friction during flow, which cannot be neglected due to the typical high viscosity of polymeric materials.

To incorporate temperature effect into the viscosity model, the Bird–Carreau–Arrhenius model was implemented based on the existing Bird–Carreau [36] model from OpenFOAM. This model is divided into two parts:

1. The calculation of the temperature shift factor (*a_T_*), which is given as follows:

(2) $$a_{T}=e^{\left[\frac{E}{R}\left(\frac{1}{T+273.15}-\frac{1}{T_{0}+273.15}\right)\right]}$$

where E is the activation energy, R the universal gas constant, and T and $T_{0}$ the temperature and the reference value, respectively.

2. Then, fluid viscosity, η is calculated as follows:

(3) $$\eta (\dot{\gamma },T)=a_{T}\left(\eta_{\infty }+\frac{\left(\eta_{0}-\eta_{\infty }\right)}{{\left(1+{\left(a_{T}\lambda \dot{\gamma }\right)}^{2}\right)}^{\frac{1-n}{2}}}\right)$$

where $\eta_{0}$ and $\eta_{\infty }$ are the Newtonian-plateau and infinite shear rate viscosities, λ is a model constant, n is the power index defining the viscosity slope in the shear-thinning region, and $\dot{\gamma }$ is the shear rate.

**Figure 1:**
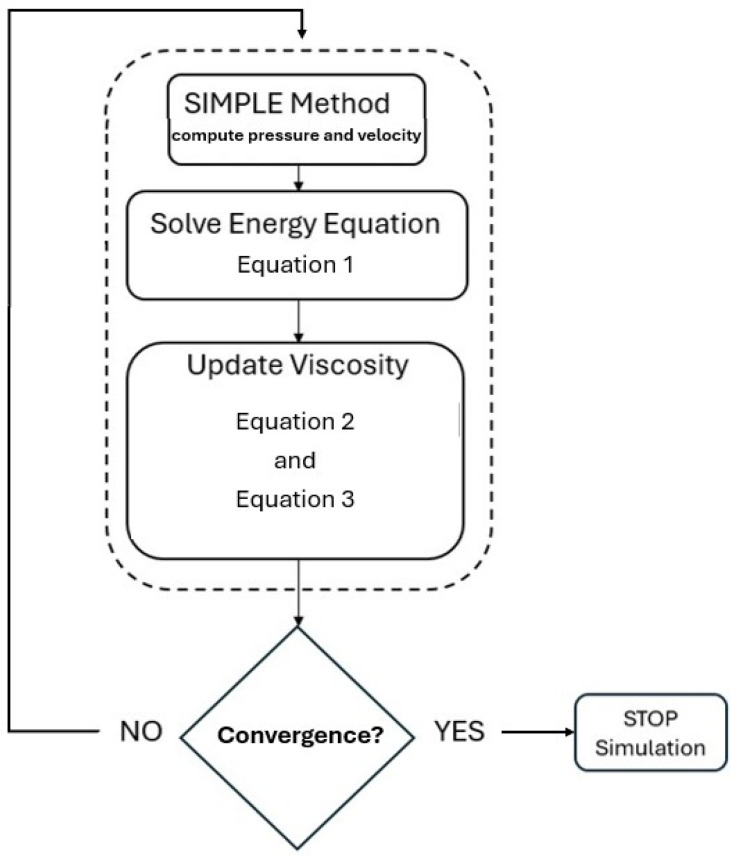
Modeling approach flowchart.

With the addition of Equations (1)–(3), the flowchart of the developed modeling code is provided in Figure 1.

The flowchart illustrates the three main steps of the calculation procedure within the developed numerical model. First, the SIMPLE method computes the pressure and velocity fields, followed by solving the energy equation (Equation (1)), which considers viscous dissipation. The resulting temperature field is then used to update the fluid’s viscosity using Equations (2) and (3), through a two-part model: an Arrhenius-type temperature shift factor and a shear-rate dependent formulation. These updated viscosity values are fed back into the momentum and energy equations, creating a fully coupled loop. The iteration continues until convergence criteria are met, based on the stabilization of the objective function described in Section 2.3. If convergence is reached, the simulation stops; if not, the loop repeats until it is achieved.

### 2.2. heatConvection Boudary Condition

To accurately represent the thermal regulation method used in the extrusion line, which controls the temperature of a thermal fluid surrounding the flow channel, a new boundary condition named *heatConvectionBC* was implemented.

To achieve this, at the relevant walls, the heat flow by convection to the thermal fluid and the heat conduction in the polymer must be equal, as follows:

(4) $$h(T_{wall}-T_{tf})=-k\nabla T$$

where h is the heat convection coefficient, $T_{wall}$ is the wall temperature, $T_{tf}$ is the thermal fluid temperature (water in this case acting as external source for controlling the temperature), k the polymer thermal conductivity, and ∇T the gradient of the temperature in the polymer. The latter can be discretized as follows:

(5) $$\nabla T=\frac{T_{wall}-T_{cell}}{d}$$

where $T_{cell}$ is the temperature at the center of the computational near the wall, and d is the distance between the center of cell and the boundary cell face normal to the face.

Combining Equations (4) and (5), the final equation for heat transfer is given as follows:

(6) $$h(T_{wall}-T_{tf})=-k(\frac{T_{wall}-T_{cell}}{d})$$

Equation (6) can be rearranged to obtain $T_{wall}$ as follows:

(7) $$T_{wall}=\frac{kT_{cell}+dhT_{tf}}{dh+k}$$

Finally, replacing the expression of $T_{wall}$ from Equation (7) into Equation (5), the final boundary condition governing the equation is obtained:

(8) $$\nabla T=\frac{hT_{tf}-hT_{cell}}{dh+k}$$

Equation (8) was implemented in the new boundary condition *heatConvectionBC* in OpenFOAM. To utilize it, the user must define the parameters h, $T_{tf}$, and k while the parameter d is calculated by OpenFOAM, as function of the boundary cell geometry, and $T_{cell}$ is calculated during the run.

### 2.3. Performance Quantification

**Figure 2:**
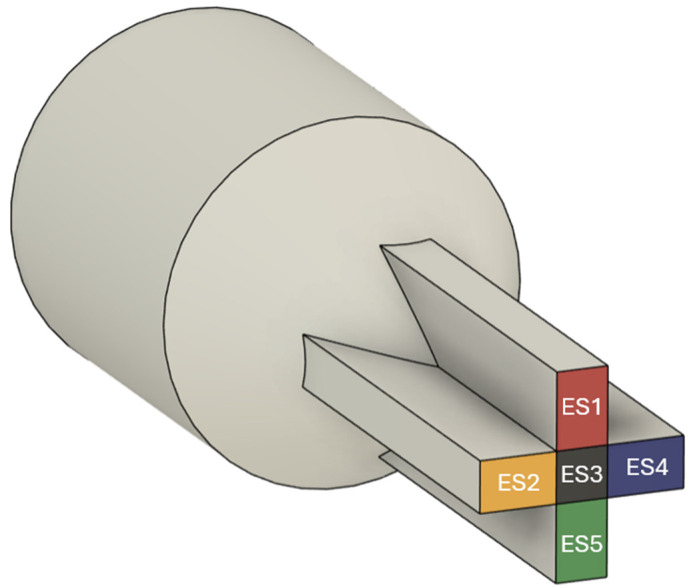
Division of an illustrative flow channel outlet into Elemental Sections (ESs).

For optimization purposes, to monitor the flow distribution, the outlet cross-section of the flow channel must be divided into Elemental Sections (ESs), as illustrated in Figure 2 for an illustrative cross-shaped profile.

This segmentation is essential because it enables an independent assessment of flow rate and velocity across different outlet regions, capturing variations that would otherwise be averaged out in a global analysis. By dividing the outlet into these regions, it becomes possible to quantify the degree of imbalance in the actual flow distribution, which directly correlates with the negative impacts of post-extrusion defects, such as warping and uneven swelling, that induce dimensional deviations. Therefore, a larger number of elemental sections are desired in this optimization framework, as they lead to a more accurate analysis of results, providing deeper insights into identifying critical zones and localized velocity non-uniformities.

To achieve faster convergence, and following the strategy proposed by Vidal et al. [26], a convergence criterion was implemented based on the stabilization of the total objective function value (see Equation (12)). The calculation was assumed to converge when the total objective function is within a tolerance of 1%.

### 2.4. Optimization Framework

**Figure 3:**
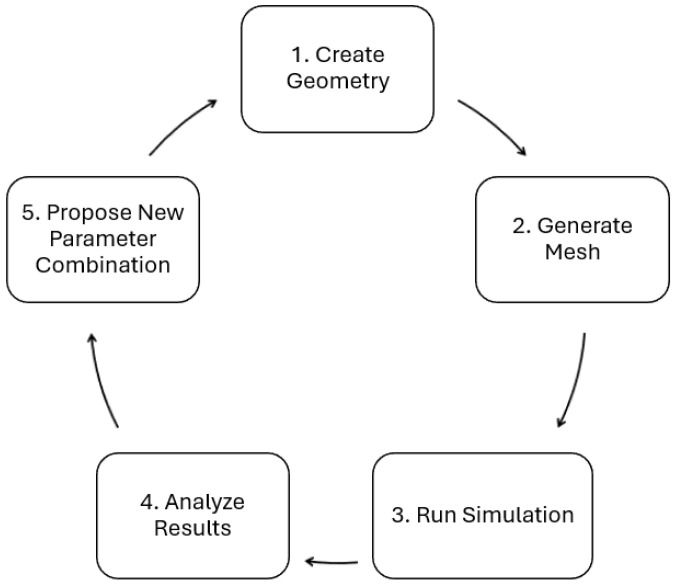
Optimization framework flowchart.

The optimization framework developed in this work integrates the extrusion die simulation in OpenFOAM [32]—an open-source computational library—with an automatic geometry update method in FreeCAD [33]—an open-source 3D CAD modeler. A complex framework composed of Python [37] version 3.8.10 codes and Bash [38] scripts was created to automatically perform preprocessing, run, and postprocessing tasks for the simulation in an iterative manner, facilitating communication between OpenFOAM simulation results and the CAD modeling of a new geometry based on the analyzed results. The optimization framework follows five main steps, illustrated in Figure 3.

**Figure 4:**
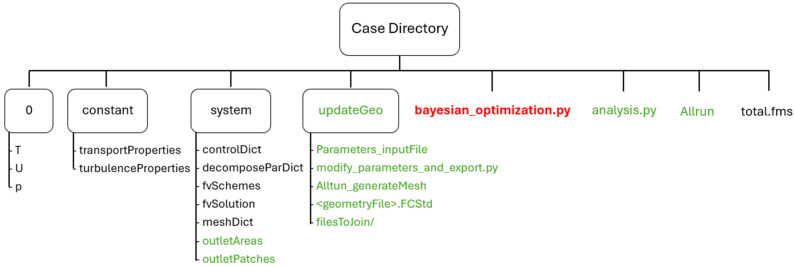
Typical simulation case folder and file organization, where files detailed in green and red represent additional dictionaries implemented for the optimization framework, while black ones represent common dictionaries for typical simulation cases in OpenFOAM.

A typical simulation case file and folder organization is illustrated in Figure 4. Folders and files detailed in green and red represent additional dictionaries implemented and required for the optimization framework, while directories and files in black represent common dictionaries needed for typical simulation cases in OpenFOAM.

In addition to the required common directories for an OpenFOAM case, such as *0*, *constant*, and *system* directories, the framework must include an additional directory named *updateGeo*. The *0* directory holds the initial and boundary conditions for the relevant unknown fields, temperature T, velocity U, and pressure p. The *constant* directory contains files related to material properties and general problem characteristics, while the *system* directory holds dictionary files responsible for controlling and specifying the simulation setup. Additional dictionary files in the *system* directory named *outletAreas* and *outletPatches* are implemented for convergence criteria purposes, as described in Section 2.3, containing die outlet information such as the number of ESs and their respective areas.

The additional *updateGeo* directory refers to the implementation of Steps 1 and 2 of the optimization framework (see Figure 3). This directory must contain all dictionary files related to updating the CAD file geometry <*geometryFile*>. *FCStd*, the file *Parameters_inputFile*, the *modify_parameters_and_export.py* code, and *Allrun_generateMesh* script. An additional directory named *filesToJoin* must also be present, containing the STL surface files for each group of geometry patches, which are automatically generated by the Python code during the optimization process.

Returning to the main case directory, an additional *Allrun* script runs the simulation (Step 3 of the optimization framework, Figure 3), while another *analysis.py* code is responsible for Step 4, calculating the pressure drop and velocity uniformity at the die exit to provide insight into the ongoing optimization procedure. The *total.fms* file contains geometry information for meshing purposes, which is common when using the *cfMesh* v1.1 [39] OpenFOAM utility. Lastly, the additional *bayesian_optimization.py* code represents the main code of the optimization framework. This code is responsible for executing all five steps of the optimization framework iteratively and must also be fed with process information such as desired objective functions, the range of each design parameter imposing the range of values the optimizer can explore, the declaration of the Bayesian optimization algorithm and function, the number of performed simulations, weight factors for each objective function, and the number of optimization iterations to be performed. The optimization loop, programmed in the Python code *bayesian_optimization.py*, serves as the controller for the entire optimization framework, automatically and iteratively executing the steps of updating geometry parameters, generating mesh, running CFD simulation, analyzing results, and proposing new parameter combinations.

Once the main code is launched, the process begins with an initial CAD geometry generated by the Python code *modify_parameters_and_export.py*, based on a parameter combination stored in the input file *Parameters_inputFile*. Along the optimization loop, the parameter combination is determined by the optimizer using results from previous simulations. If an existing dataset is stored in an optional file named *InputSimulationData*, it is used by the optimizer to impose the first parameter combination. If no prior dataset is provided, the optimizer initializes the first simulation using the midpoint values for all parameters, as defined in the search space within the Phyton code (*bayesian_optimization.py*). Subsequently, the *Allrun_generateMesh* Bash script is used to mesh the geometry with the OpenFOAM mesh generator utility named *cfMesh* [39]. Later, the simulation is launched through another Bash script named *Allrun*, which also handles other preprocessing tasks such as mesh-decomposing (for parallel runs) the case and calculating the area of the different ESs, crucial for postprocessing tasks and accurately calculating the flow rate for the implemented convergence criteria (see Section 2.3). After the simulation is completed, the Python code *analysis.py* calculates the pressure drop in the channel and the velocity uniformity at the flow-channel outlet, computing the standard deviation of the different elemental sections and identifying their uniformity. The calculated values of pressure-drop and velocity uniformity are stored in the output file named *BayesianOptimization.txt*, labeled with the employed parameter combination. Lastly, a Bayesian optimization algorithm from Scikit-Optimize [40] is used, leveraging the *gp_minimize* function [41] to propose a new parameter combination according to the simulated results, aiming to find the optimal parameters that are expected to yield the best value for the objective function under study. The proposed new parameter combination is then defined in the file *Parameters_inputFile*, prompting the CAD geometry update by re-running the Python code *modify_parameters_and_export.py*. The process is then repeated iteratively for a user-specified number of trials, defined in the main *bayesian_optimization.py* code.

Each of the different framework steps is detailed in the following subsections.

#### 2.4.1. Geometry

**Figure 5:**
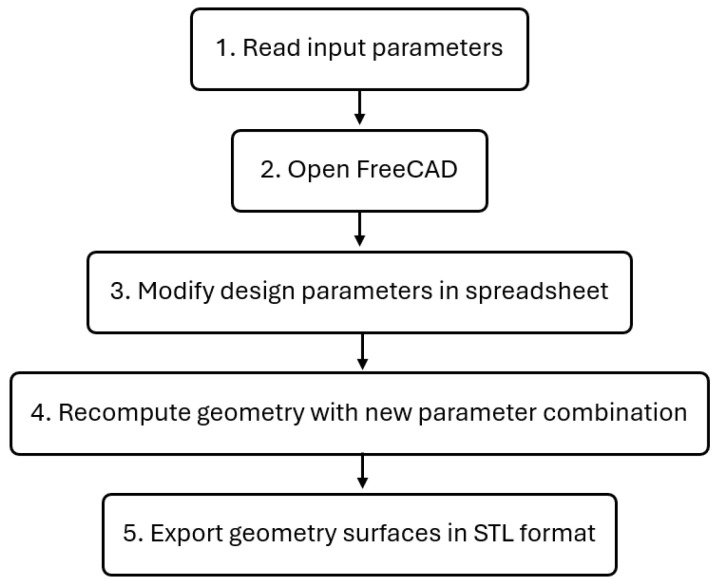
Geometry update flowchart.

The geometry used in this optimization framework is generated by FreeCAD v1 [33], an open-source 3D parametric modeling software with a built-in Python scripting environment, allowing for task automation. To leverage this feature and streamline the geometry creation process, a Python script was developed to update the geometry. The flowchart of the code structure is illustrated in Figure 5.

This code opens the 3D model file in FreeCAD and modifies the dimensions of the geometry parameters in a sketch design based on values from the input file. These parameters serve as control points to define the trial geometry. To enable this automation, a spreadsheet was embedded within the FreeCAD 3D model file structure, with different cells assigned aliases for each design parameter. When the Python script executes, it automatically updates the spreadsheet values with those from the input file in the OpenFOAM case directory, modifying the geometry accordingly. After creating the new geometry with the updated parameter combinations, the Python code exports the surfaces as separate STL files based on their boundary representation.

#### 2.4.2. Mesh

**Figure 6:**
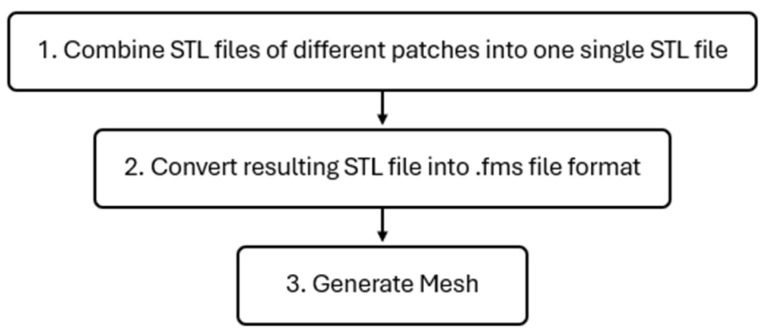
Mesh generation flowchart.

After creating the geometry, mesh generation was accomplished using a bash script to run *cfMesh* v1.1 [39], an automatic mesh generator utility provided by OpenFOAM. The mesh generation flowchart is illustrated in Figure 6.

Since the preferred geometry file format for *cfMesh* [39] is *.fms*—due to its structure designed to hold relevant information for setting up a meshing job, such as patch names and their respective boundary types for OpenFOAM—the script’s first task is to combine the STL files of the different patches into a single STL file containing the entire geometry. To do this, the bash script merges the STL files into one *total.stl* file and then uses the *surfaceFeatureEdges* [42] utility from OpenFOAM to convert the *total.stl* file into a *total.fms* file. Finally, the bash script runs the *cartesianMesh* utility [43] from *cfMesh* to generate a 3D mesh, which is stored in the simulation case directory.

#### 2.4.3. Run Simulation

**Figure 7:**
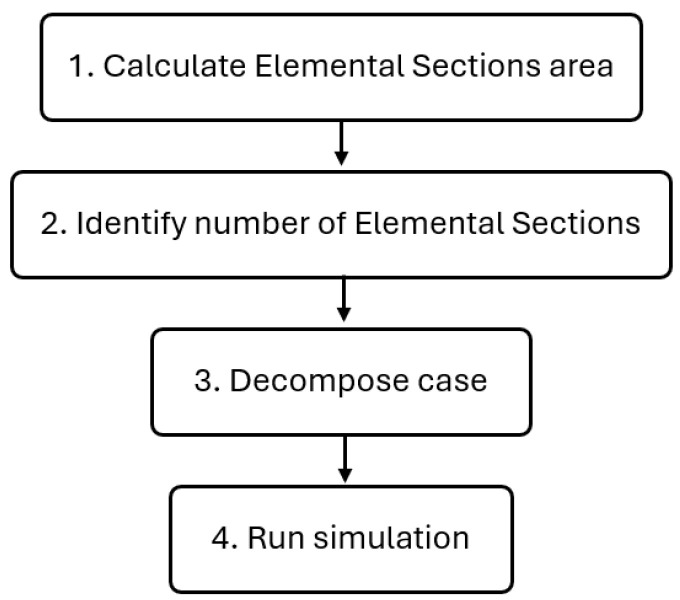
Running simulation flowchart.

Once the computational mesh has been generated, the CFD simulation step is started by launching a Bash script responsible for running and automating several crucial preprocessing, execution and postprocessing steps in the optimization framework. The flowchart of this process is illustrated in Figure 7, which aims at ensuring that the case is correctly set up, executed, and the results are properly extracted for the following 4th (Analyze Results) and 5th (Propose New Parameter Combination) steps of the framework (see Figure 3).

The first step in the preprocessing phase involves calculating the area of the various ESs at the flow channel outlet using the *patchIntegrate* [44] OpenFOAM utility. Since meshing a CAD model can slightly alter its original size due to inherent constraints, this step is crucial for accurately determining the area of each ES for the simulation mesh. Correctly defining these areas will ensure accurate flow rate calculations for each elemental section and their corresponding average velocities, thereby assessing and ensuring the reliability of the convergence criteria.

As a second preprocessing step, the script identifies the number of ESs in the mesh, which is necessary for performing postprocessing tasks like calculating velocity uniformity. Finally, in the third and last preprocessing step, due to the computational intensity of these simulations, the script decomposes the case into multiple subdomains using the *decomposePar* OpenFOAM utility, which is essential for parallel runs.

Once the preprocessing steps are complete, the script executes the CFD simulation using the *viscousSimpleFoam* solver (see Section 2.1). The simulation continues until the convergence criterion is met, ensuring that the flow field is well established before extracting results (see Section 2.3). Finally, after the simulation concludes, the script moves to the postprocessing step, which is the fourth step of the optimization framework (see Figure 3) and is responsible for analyzing the results. This step is detailed in the next subsection.

#### 2.4.4. Analyze Results

**Figure 8:**
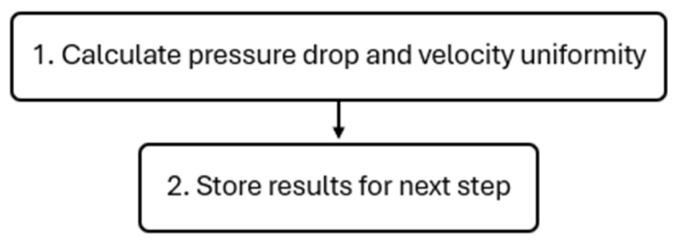
Results analysis flowchart.

Once the flow field stabilizes according to the predefined convergence criteria and the CFD simulation is complete, the next step of the optimization framework involves extracting and analyzing the simulated results. This process occurs in two steps, as shown in Figure 8.

The script mentioned in the previous subsection also automatically performs the necessary steps, ensuring that the computed data are processed correctly and formatted for the subsequent optimization step. The results analysis focused on two critical performance indicators of the extrusion simulation, which will later serve as objective functions in the Bayesian optimization:

1—Pressure Drop (ΔP):

This quantity is correlated with the energy required to create flow within the extrusion die flow channel, representing a key factor in the extrusion process as it directly influences the resources needed to extrude the material. Since the pressure at the flow channel outlet is set to 0 Pa to represent atmospheric pressure, the total pressure drop is simply the inlet pressure.

$$\left(\Delta P=P_{inlet}\right)$$

To this end, the pressure value at the flow channel inlet is calculated by OpenFOAM for use as input data in the next step, which aims to propose a new parameter combination in the fifth step of the optimization (see Figure 3).

2—Velocity Uniformity ($U_{unif}$):

We quantify the uniformity of average velocity across different ESs of the die outlet cross-section, which is crucial for the extrusion process as it directly affects the quality of the extruded profile. A non-uniform velocity profile at the die outlet leads to defects and distortions in the extruded material, as an unbalanced flow means the material does not emerge from the flow channel at the average velocity across all ESs.

To achieve this, the Python code loops through each ES at the die outlet, extracting the corresponding average velocity values calculated by OpenFOAM. Subsequently, the standard deviation ($\sigma_{U}$) of the ESs is calculated:

(9) $$\sigma_{U}=\sqrt{\frac{\overset{N}{\underset{i=1}{\sum }}{\left(U_{i,avg}-U_{total,avgMean}\right)}^{2}}{N}}$$

where $U_{i,avg}$ is the average velocity of ES *i*, N is the total number of ES, and $U_{total,avgMean}$ is the global mean velocity given as follows:

(10) $$U_{total,avgMean}=\frac{\overset{N}{\underset{i=1}{\sum }}U_{i,avg}}{N}$$

Finally, the dimensionless velocity uniformity performance indicator is calculated as follows:

(11) $$U_{unif}=1-\frac{\sigma_{U}}{U_{total,avgMean}}$$

which quantifies uniformity on a scale from 0 to 1, with 0 representing the worst scenario and 1 indicating a completely balanced flow. The calculated value is then stored and will be used as input for the next step, which proposes new parameter combinations, as detailed in the following subsection.

#### 2.4.5. Propose New Parameter Combination

After analyzing the simulation results, the next and final step in the optimization framework is to propose a new set of parameters for the extrusion die geometry to restart the optimization loop. This stage automatically determines the next parameter combination based on the analyzed results using the Bayesian optimization algorithm from Scikit-Optimize (Skopt) [40], specifically leveraging the *gp_minimize* function. The proposal of new parameter values aims to balance improving the best-found solutions and exploring new regions of the search space.

**Figure 9:**
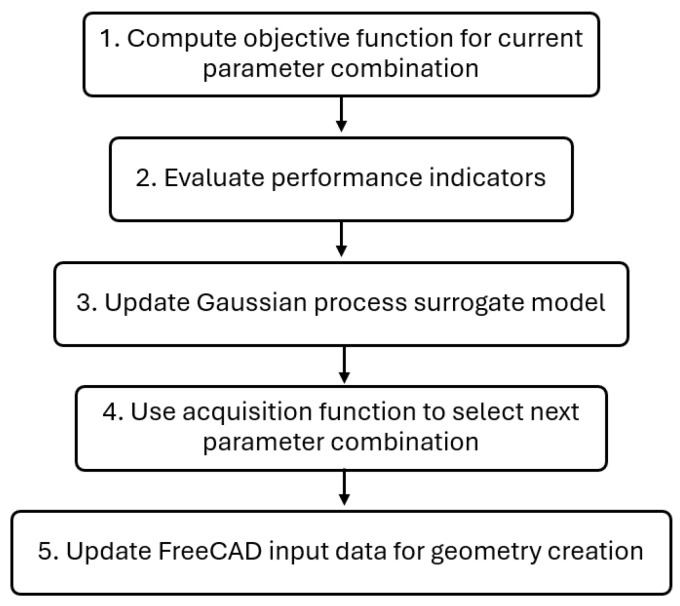
Proposal of parameter combination flowchart.

The flowchart for proposing the new parameter combinations is illustrated in Figure 9.

To determine the next parameter combination, the optimizer evaluates the performance of the current set of parameters by computing for the simulated trial the optimization objective function ($F_{objOpt}$):

(12) $$F_{objOpt}=w_{1}\Delta P_{norm}+w_{2}(1-U_{unif})$$

The obtained values aims at balancing the normalized pressure drop ($\Delta P_{norm}$) and velocity uniformity ($U_{unif}$), given by Equation (11), with weighting factors $w_{1}$ and $w_{2}$, which are predefined by the user. To this end, the optimizer’s role is to reach the minimum value for the employed objective function, where values closer to 0 indicate better results while those closer to 1 indicate the worst results. Since minimizing the objective function was the target, and the calculated velocity uniformity already presented a 0 to 1 scale (Equation (11)), pressure drop was also normalized to ensure that both metrics contributed proportionally to the calculated objective function. To this end, the code was developed to calculate the maximum ($\Delta P_{\max}$) and minimum ($\Delta P_{\min}$) pressure drops values among current and previous simulations results, and calculate the normalization of the pressure drop of the current set of parameters, as follows:

(13) $$\Delta P_{norm}=\frac{\Delta P-\Delta P_{\min}}{\Delta P_{\max}-\Delta P_{\min}}$$

Once the objective function is computed, the optimizer uses the *gp_minimize* function from Scikit-Optimize to perform Bayesian optimization based on Gaussian process regression [45]. This approach is particularly useful for computationally expensive functions, such as CFD simulations, as it builds a predictive model of the objective function based on previously evaluated combinations. In other words, the *gp_minimize* function estimates how the objective function will behave for different parameter values, proposing a new set of parameters that is likely to improve the current best result, rather than suggesting a random new set. The model is updated with new data after each simulation, increasing the dataset and refining its predictions, progressively leading to the optimal parameter set. In the optimization framework presented in this work, the acquisition function—responsible for selecting the next parameter combination to try—was defined as Expected Improvement (EI), which balances exploration (searching unexplored regions of parameter space) and exploitation (refining already explored regions), ensuring an efficient search for the optimal combination of parameters to achieve the minimum value for the objective function.

## 3. Case Study

**Figure 10:**
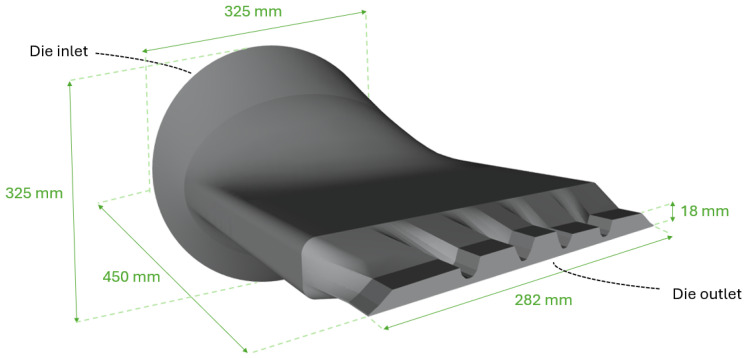
Extrusion die geometry employed.

To validate the performance and applicability of the proposed optimization framework, two case studies were conducted using representative geometries of an extrusion die for a tire manufacturing. The first consists of a more complex geometry, as illustrated in Figure 10, with varying thicknesses at the die outlet section, while the second study consisted of a simpler geometry with constant thicknesses. To avoid extending the text, for interested reader, the second optimization case study is presented in the Supplementary Material. In both cases, the illustrated flow channel can be manufactured with a typical method employed for extrusion dies, wire EDM.

In this specific and illustrative problem, the optimization aimed at:

- Maximizing velocity uniformity across the elemental sections (ES) to ensure a homogeneous extrudate profile;
- Minimizing the overall pressure drop along the channel, reducing energy consumption;

For the optimization loop, these performance metrics were combined into a single objective function, as described in Section 2.4.5, with greater emphasis placed on velocity uniformity. Accordingly, the pressure drop and velocity uniformity weighting factor were defined as $w_{1}=0.2$ and $w_{2}=0.8$, respectively. The full optimization cycle—including geometry update, mesh generation, simulation, postprocessing, and parameter selection—was executed automatically using the developed framework, demonstrating its robustness and efficiency for practical engineering applications.

To reduce computational cost (time), the convergence criteria (Section 2.3) tolerance value for each simulation was set to 10^−3^ over 100 consecutive iterations. With that, simulations were stopped when performance indicators based on elemental sections flow rate variations fell below 10^−3^ for 100 consecutive iterations, providing a reliable measure of flow stabilization.

The computational mesh employed was selected based on a detailed mesh sensitivity study that is described in the Supplementary Materials.

### 3.1. Extrusion Die Geometry

The geometry used in this work consisted of an extrusion die channel with a tire tread cross-section as the outlet shape, as shown in Figure 10. The channel featured an inlet with an area of approximately 83,000 mm^2^, converging downstream to the tire tread shape at the outlet, with an area of approximately 3300 mm^2^.

**Figure 11:**
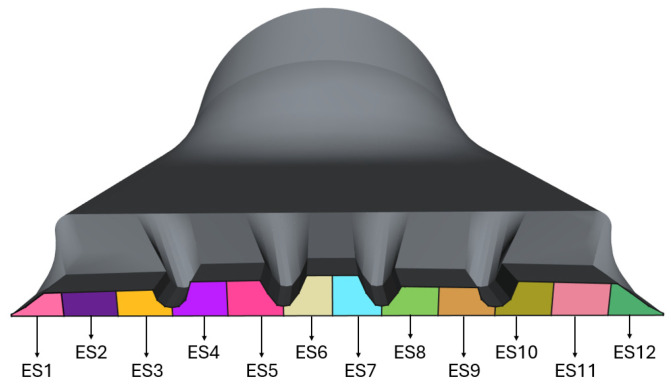
Division of the extrusion die flow channel outlet into Elemental Sections (ES).

To analyze the flow distribution and improve velocity uniformity at the extrusion die flow channel outlet, the outlet was divided into 12 different ES, as illustrated in Figure 11. This approach was necessary to utilize the convergence criteria described in Section 2, identifying the flow rate and average velocity for each section. This enabled accurate determination of the flow distribution and velocity profile at the flow-channel outlet, providing insights into potential modifications to enhance velocity uniformity. To increase case complexity, the outlet section was designed with varying thicknesses to challenge the optimization framework to find the best parameters configuration for flow balancing. For instance, outlet heights in ES1 to ES3 are smaller than in ES10 to ES12, while height in ES4 and ES5 are greater than ES8 and ES9. The center of the outlet was designed to be the thicker section, in ES6 and ES7.

**Figure 12:**
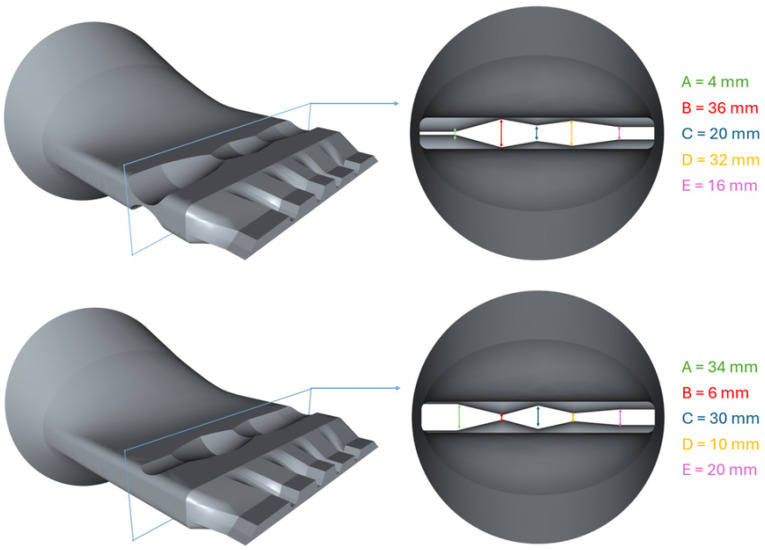
Location of the flow obstruction in the extrusion die flow channel, and example of different combinations of the Design Parameters and their corresponding geometry.

To allow controlling the flow distribution, a flow obstruction was inserted in the pre-parallel zone of the extrusion die flow channel—a region commonly used to enhance flow conditions before reaching the outlet section, as illustrated in Figure 12.

The flow obstruction was designed to influence the flow direction along the channel and control the flow distribution at the extrusion die outlet. For automation purposes, this obstruction was fully parameterized with five design parameters named A, B, C, D and E, which could vary between 2 mm and 40 mm. That positive minimum value assures that the top and bottom surfaces do not intersect, and that the obstruction regions can be also manufactured by Wire EDM. As shown in Figure 12, the referred parameters serve as control points that define the obstruction’s cross-section shape, allowing for the generation of different geometries that guide the material flow in specific directions and allow looking for and improved uniformity at the flow channel outlet. To avoid sharp edges and enhance flow smoothness and machinability, the obstruction’s shape follows a smooth design defined by a spline.

### 3.2. Material Properties

The material employed in the simulation was a Generalized Newtonian Fluid (GNF), leveraging the Bird–Carreau–Arrhenius model implemented as described in Section 2.1. Actual rubber material is typically employed in tire manufacturing and the properties were provided by Michelin Ladoux, this work industrial partner, as shown in Table 1.

### 3.3. Boundary Conditions

To accurately replicate the extrusion conditions and experimental process at Michelin Ladoux premises, it is essential to define the correct boundary conditions and case setup for the CFD simulation. A volumetric flow rate of 2.898 cm^3^/s was set for the inlet patch with the *flowRateInletVelocity* Boundary Condition (BC), utilizing a *table* option from OpenFOAM [46] that gradually increases the volumetric flow rate from 0.01 cm^3^/s to 2.898 cm^3^/s over the first 50 s. This approach allows preventing numerical instabilities caused by high velocity gradients at the start of the simulation. At the outlet patch, a pressure of 0 Pa was imposed to represent atmospheric pressure, while a null gradient was applied for the velocity field using the *zeroGradient* BC, assuming as usual fully developed conditions. These are typically the only possible conditions for outlets in this type of process [1], as no other flow information is available at this location. For the walls, adherence was assumed, leading to the application of a no slip boundary condition for the velocity field, with *zeroGradient* applied for the pressure field.

For the temperature field, the material was set to enter the channel at a constant temperature of 90 degrees Celsius, while *zeroGradient* was applied to the outlet. On the walls, to accurately represent thermal regulation through heat exchange between the system and external conditions, a new boundary condition named *heatConvectionBC* was implemented.

Table 2 shows the employed boundary conditions for Inlet, Outlet (ES1 to ES12) and walls.

### 3.4. Case Setup

In the main *bayesianOptimisation.py* code, the Bayesian optimization loop was set to perform 25 iterations, using the *gp_minimize* function from Scikit-Optimize. Each iteration included a complete optimization cycle: geometry update, meshing, CFD simulation, and extraction of performance metrics. Thus, a total of 25 simulations were conducted to explore and refine the geometric parameters A, B, C, D and E, with the goal of minimizing the objective function defined in Section 2.4.5.

The acquisition function was defined as Expected Improvement (EI), which aimed to balance exploration and exploitation of the search space to identify the optimal parameter combinations within the 25 iterations. The search space (parameter range) was defined with a lower bound of 2 mm and an upper bound of 40 mm for all five parameters.

As an initial dataset, a simulation was previously run with no obstructions in the channel (A = 40 mm, B = 40 mm, C = 40 mm, D = 40 mm, E = 40 mm) to provide the algorithm with initial information for the optimization loop, as shown in Table 3, which also provides the reference values for the total pressure drop and velocity uniformity.

## 4. Results and Discussion

**Figure 13:**
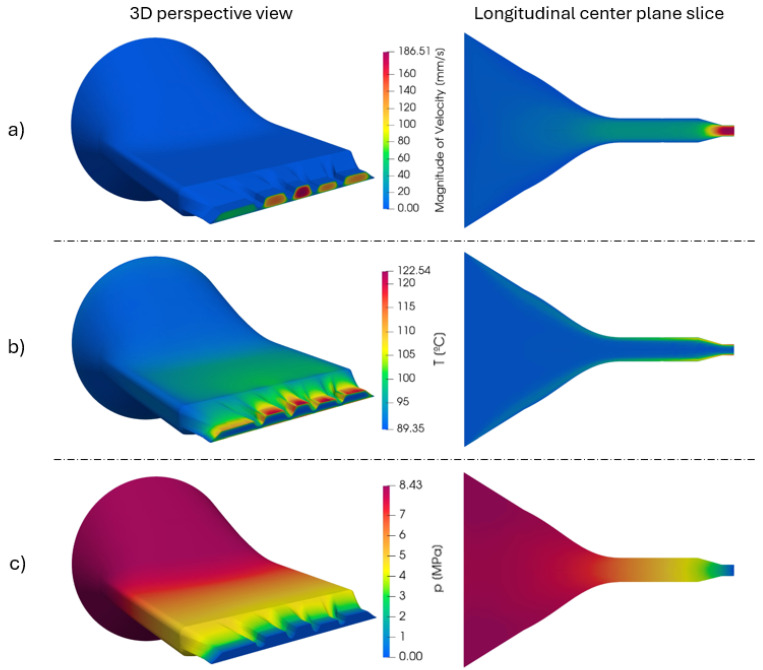
Simulation results of (**a**) velocity, (**b**) temperature, and (**c**) pressure fields for initial dataset geometry. Left column: full geometry 3D view; right column: longitudinal center plane slice illustrating internal flow development.

Simulation results for the initial die geometry, which served as the dataset for the starting point of the optimization process, are presented in Figure 13. The velocity field (Figure 13a) reveals a very unbalanced flow at the outlet, presenting higher velocities concentrated near the center of the outlet channel (ES6 and ES7) and significantly lower velocities near the lateral sections, especially at the left side (ES1 to ES3). The temperature field (Figure 13b) shows an increase in temperature towards the outlet, particularly concentrated in regions with higher velocity. This behavior is consistent with the viscous heating expected in regions of greater shear rate. Finally, the pressure field (Figure 13c) exhibits a gradual decrease along the flow direction, with a total pressure drop of approximately 8.4 MPa.

The sequence of simulations conducted during the optimization process is shown in Table 4, along with the parameter combinations proposed by the Bayesian optimization algorithm and their corresponding results for pressure drop ΔP and velocity uniformity $U_{unif}$. The objective function was calculated by dynamically normalizing the pressure drop values and combining them with the uniformity results using predefined weighting factors of 0.2 and 0.8, respectively.

**Figure 14:**
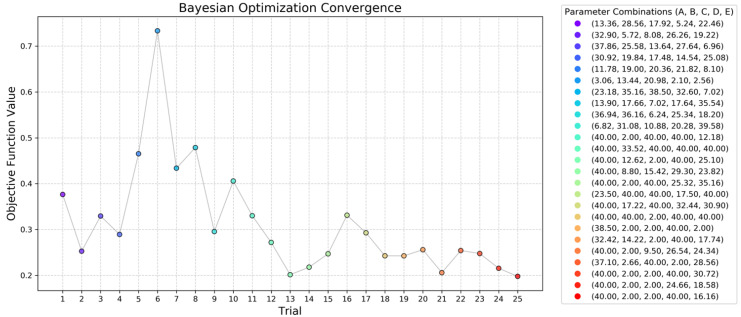
Objective function evolution over the optimization trial.

The entire optimization loop took approximately 50 h to be concluded, with an average time of 2 h per simulation, in a desktop computer with an Intel Core i7-12700K processor (12 cores, 20 threads, up to 5.0 GHz). To expedite the process, cases were decomposed and run using 12 cores. Depending on the complexity of each geometry, simulation times ranged from 1 to 3 h until the convergence criteria were met, which was achieved within an average of 200 time-steps. The table shows that the optimizer explores a wide range of parameter combinations in the initial iterations, yielding varying performance levels. The objective function values decrease progressively as the optimizer converges toward better configurations. This convergence behavior and the improvements in velocity uniformity are discussed further in the following paragraphs, supported by graphical analysis. Figure 14 presents the evolution of the objective function over the optimization iterations, illustrating the convergence behavior throughout the process.

The initial iterations show moderate variation in the objective function values, reflecting the exploration phase of the Bayesian optimizer, during which the algorithm sampled a wide range of regions in the design space, including those significantly different from the input dataset. A significant peak was observed at Trial 6, marking the highest objective value (0.7333), corresponding to a configuration with a high pressure drop (12.0722 MPa) and poor velocity uniformity (0.3334). As the process progressed, the optimizer refined its search, transitioning to the exploitation phase where it focused on the most promising areas of the design space. A clear downward trend in the objective function was observed, particularly from Trial 10 onward. The lowest objective value (0.2001) was reached at the final iteration (Trial 25), corresponding to the most balanced combination of pressure drop and velocity uniformity found in this study.

**Figure 15:**
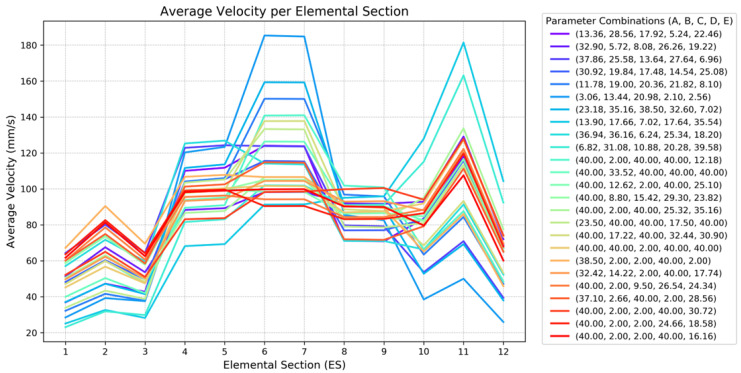
Elemental Sections average velocity for different parameter combinations.

The evolution in velocity distribution at the outlet is shown in Figure 15, where the average velocity across the 12 Elemental Sections (ESs) is plotted for each simulated geometry.

In the early iterations, most configurations displayed significant velocity non-uniformity across the outlet, with ES6, ES7 and ES11 often exhibiting over- or under-velocity compared to the remaining sections. These profiles are represented by the more irregular curves, as seen in dark blue (Trial 6, with the greatest velocity in ES6 and ES7) and light blue (Trial 8, with the greatest velocity in ES11) lines in the graph. As optimization progressed, the outlet profiles became increasingly uniform and centered, as shown in the final iterations, where velocity differences across sections were minimized.

The optimal performance was obtained with geometry of Trial 25:

- A = 40.00 mm, B = 2.00 mm, C = 2.00 mm, D = 40.00 mm, E = 16.16 mm;
- Objective function value: 0.2001;
- Velocity uniformity: 0.8137;
- Pressure drop: 9.3612 MPa.

**Figure 16:**
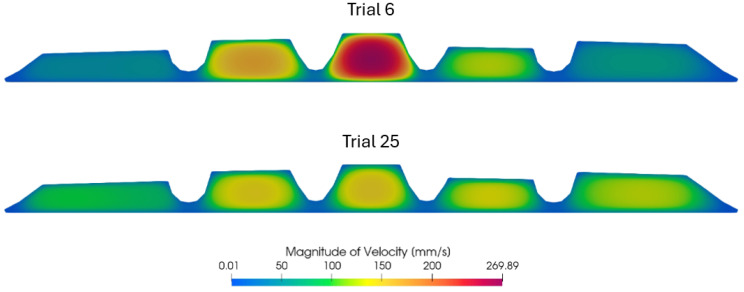
Die outlet velocity field comparison for Trial 6 (**top**) and Trial 25 (**bottom**) of the optimization loop.

To demonstrate the flexibility and validation of the optimization framework, Figure 16 shows the velocity magnitude distribution at the die outlet for two selected geometries evaluated during the optimization process: Trial 6, the worst-performing configuration, and Trial 25, the optimized geometry with the lowest objective function value. In Trial 6, the flow is significantly non-uniform, with higher velocities concentrated in the central ESs and stagnation near the lateral ESs, leading to a noticeable imbalance across the outlet. In contrast, Trial 25 exhibits a much more uniform velocity distribution across all ESs. The velocity peaks are smoother and more evenly spread across the domain, reflecting a more balanced and efficient internal flow field. This optimized flow behavior directly contributes to the improved performance indicators observed for Trial 25, including higher uniformity and a reduced pressure drop.

In addition to achieving the best objective function value within the optimization loop, the configuration found in Trial 25 outperformed the baseline geometry provided in the input dataset, which had no flow obstruction. While a jump of 11% in pressure drop was observed in the optimized geometry (9.36 MPa compared to 8.43 MPa), an astonishing enhancement of 23.9% in outlet velocity uniformity was obtained (0.8137 compared to 0.6568). Since velocity uniformity had a greater impact in the objective function due to weighting factors (0.2 for pressure drop and 0.8 for velocity uniformity), the overall objective function value improved from 0.2746 in the baseline geometry to 0.2001 in the optimized design, confirming the effectiveness of the optimization even in a multi-objective trade-off scenario. However, if the optimization had focused solely on velocity uniformity, the configuration from Trial 24 would have been selected as the optimal geometry, as it yielded the highest uniformity among all simulations (0.8200).

This observation underscores the importance of the weighting strategy in multi-objective optimization and the framework’s flexibility in adapting to different design priorities. Additionally, it confirms that adjusting the internal geometry through the A, B, C, D, and E parameters effectively influences flow distribution, validating the framework’s capability to guide the design process toward optimized solutions in a fully automated manner.

## 5. Conclusions

In this study, a fully automated optimization framework that can be applied to the design of extrusion dies was developed, specifically targeting improvements in outlet velocity uniformity and reductions in pressure drop. The framework integrates CAD model generation in FreeCAD, CFD simulation in OpenFOAM, and Bayesian optimization using Scikit-Optimize, creating a pipeline capable of exploring complex design spaces with minimal manual intervention.

A case study was conducted on a 3D extrusion die geometry, featuring a representative outlet shape utilized in tire manufacturing, provided by the industrial partner, Michelin. The flow obstruction profile at the pre-parallel zone was parameterized through five geometric variables (A, B, C, D, and E), each controlling the die cross-section shape to influence flow conditioning. The simulations accounted for non-Newtonian fluid behavior, modeled as a Generalized Newtonian Fluid (GNF) using the Bird–Carreau model, with temperature dependence described by the Arrhenius law, accurately reflecting the rheology of rubber compounds used in tire extrusion processes.

The optimization aimed to achieve an optimal balance between outlet velocity uniformity and pressure drop, utilizing a weighted objective function. After 25 automated iterations, the framework converged to an optimal geometry with an objective function value of 0.2001, surpassing the input dataset configuration (without any channel obstruction) and alternative candidates within the optimization loop. The results demonstrated that targeted geometric modifications, even with a limited number of control parameters, can lead to significant improvements in flow uniformity and pressure efficiency.

The study also underscored the framework’s adaptability to diverse design goals. For example, in case study 2 presented in the Supplementary Materials, if velocity uniformity had been the sole objective, a different configuration (Trial 4) would have been favored instead of the one presenting the best trade-off between pressure drop and uniformity, highlighting the influence of the chosen weighting strategy in multi-objective optimization.

Despite these promising outcomes, certain limitations must be acknowledged. Although the design of the obstruction used in this study was relatively simple, it is important to note that in a study involving more complex geometries, the shape proposed by the optimization framework can be manufactured by wire EDM. Furthermore, although the rheological model used was advanced and suitable for this material class, the study did not consider effects such as wall slip or viscoelasticity.

Future work could extend the framework to incorporate more complex geometries, viscoelastic constitutive models, and structural or manufacturability constraints. Additionally, integrating multi-objective optimization techniques, such as Pareto front generation, would facilitate the identification of a set of equally optimal solutions under various trade-off conditions. These enhancements would further elevate the applicability and realism of the tool in industrial extrusion die design and process optimization. Experimental validation for the optimization framework is also expected to be conducted at the industrial partner’s facilities using an industrial-scale geometry at a later stage of the work.

In summary, the proposed framework has proven to be a robust and effective tool for the geometry-driven optimization of 3D extrusion dies using realistic material models. Its modular and automated structure establishes a valuable foundation for broader deployment in engineering design workflows within the polymer processing industry.

## Figures and Tables

**Table 1:** Material properties.

Property	Description	Value	Units
$\eta_{0}$	Viscosity at zero shear rate	10^7^	Pa.s
$\eta_{\infty }$	Viscosity at infinite shear rate	100	Pa.s
λ	Characteristic time	100	s
n	Power-law index	0.2	
$\frac{E}{R}$	Ratio between activation energy and the universal gas constant	1000	K
$T_{0}$	Reference temperature	330	K
α	Thermal diffusivity	1.28 × 10^−7^	m^2^/s
$c_{p}$	Specific heat capacity	1700	J/(kg.K)
ρ	Density	1150	kg/m^3^

**Table 2:** Boundary conditions.

Field	Inlet	Outlets (ES1 to ES12)	Walls
Pressure p	Null normal gradient	0 [Pa]	Null normal gradient
Velocity u	Flow rate from 0.01 [cm^3^/s] to 2.898 [cm^3^/s]	Null normal gradient	noSlip
Temperature T	363.15 [K]	Null normal gradient	*heatConvectionBC* with h = 0.25 [W/m^2^.K] $T_{tf}$ = 363.15 [K] k = 0.25 [W/m.K]

**Table 3:** Initial dataset for optimization loop.

A [mm]	B [mm]	C [mm]	B [mm]	C [mm]	ΔP [MPa]	$U_{unif}$
40.00	40.00	40.00	40.00	40.00	8.4307	0.6568

**Table 4:** Optimization loop results.

Trial	A [mm]	B [mm]	C [mm]	D [mm]	E [mm]	ΔP [MPa]	$U_{unif}$	$F_{objOpt}$
1	13.36	28.56	17.92	5.24	22.46	9.9151	0.6292	0.3782
2	32.90	5.72	8.08	26.26	19.22	9.5613	0.7591	0.2548
3	37.86	25.58	13.64	27.64	6.96	9.5098	0.6597	0.3315
4	30.92	19.84	17.48	14.54	25.08	9.5206	0.7106	0.2914
5	11.78	19.00	20.36	21.82	8.10	10.1620	0.5354	0.4668
6	3.06	13.44	20.98	2.10	2.56	12.0722	0.3334	0.7333
7	23.18	35.16	38.50	32.60	7.02	9.0088	0.4944	0.4362
8	13.90	17.66	7.02	17.64	35.54	9.7914	0.4928	0.4805
9	36.94	36.16	6.24	25.34	18.20	9.2974	0.6872	0.2978
10	6.82	31.08	10.88	20.28	39.58	9.2398	0.5455	0.4080
11	40.00	2.00	40.00	40.00	12.18	9.0725	0.6284	0.3325
12	40.00	33.52	40.00	40.00	40.00	8.4828	0.6601	0.2748
13	40.00	12.62	2.00	40.00	25.10	9.1944	0.7976	0.2039
14	40.00	8.80	15.42	29.30	23.82	9.3614	0.7887	0.2202
15	40.00	2.00	40.00	25.32	35.16	9.0329	0.7294	0.2496
16	23.50	40.00	40.00	17.50	40.00	8.7703	0.6060	0.3339
17	40.00	17.22	40.00	32.44	30.90	8.6860	0.6480	0.2956
18	40.00	40.00	2.00	40.00	40.00	8.8295	0.7207	0.2453
19	38.50	2.00	2.00	40.00	2.00	9.6116	0.7753	0.2446
20	32.42	14.22	2.00	40.00	17.74	9.4469	0.7471	0.2581
21	40.00	2.00	9.50	26.54	24.34	9.4036	0.8066	0.2082
22	37.10	2.66	40.00	2.00	28.56	9.5401	0.7559	0.2562
23	40.00	2.00	2.00	40.00	30.72	9.3470	0.7505	0.2499
24	40.00	2.00	2.00	24.66	18.58	9.7672	0.8200	0.2174
25	40.00	2.00	2.00	40.00	16.16	9.3612	0.8137	0.2001

## Data Availability

The raw data supporting the conclusions of this article will be made available by the authors on request.

## Supplementary Materials

The following supporting information can be downloaded at: https://www.mdpi.com/article/10.3390/polym17111498/s1, Figure S1: Extrusion die flow channel computational mesh; Figure S2: Extrusion die geometry employed for case study 2; Figure S3: Division of the extrusion die flow channel outlet into Elemental Sections (ES); Figure S4 Location of the flow obstruction in the extrusion die flow channel, and example of different combinations of the Design Parameters and their corresponding geometry; Figure S5: Simulation results of (a) velocity, (b) temperature, and (c) pressure fields for initial dataset geometry of case study 2. Left column: full geometry 3D view; right column: longitudinal center plane slice illustrating internal flow development; Figure S6: Objective Function evolution over the optimization trial; Figure S7: Elemental Sections average velocity for different parameter combinations; Figure S8: Die outlet velocity field comparison for Trial 5 (top) and Trial 19 (bottom) of the optimization loop, for case study 2; Table S1: Initial dataset for optimization loop for case study 2; Table S2: Optimization loop results for case study 2.
