# Supplementary material for: A Novel Approach to Automatically Balance Flow in Profile Extrusion Dies Through Computational Modeling
Source: Polymers (Basel). 2025 May 28;17(11):1498. doi: 10.3390/polym17111498 (PMC12157835; doi:10.3390/polym17111498)
Supplement: Supplementary file 1 [file polymers-17-01498-s001.zip › caseStudy2_final.pdf]

The second case study was conducted with same case setup as the case study for a more complex geometry, as described in Section 3, but with a simpler geometry as illustrated in Figure S2. Differently to the other case study, this geometry was designed with shorter channel and a constant thickness across the outlet section, thus, elemental sections contained the same height. That said, geometry optimization was also targeted to maximize velocity uniformity at the die outlet and minimize the overall pressure drop along the channel. For that, weighting factors were also defined as 0.2 and 0.8 for pressure drop and velocity uniformity, respectively, and simulation convergence criteria was set to 10^{-3} over 100 consecutive iterations.

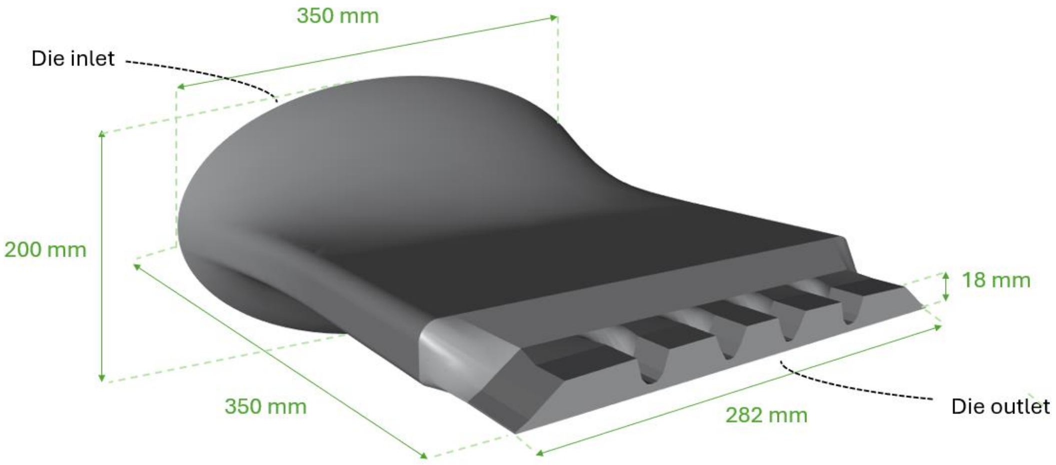

Figure S2. Extrusion die geometry employed for case study 2.

The geometry used in this study involved an extrusion die channel with a tire tread cross-section at the outlet (Figure S2), with an inlet area of 55,000 mm² converging to an outlet area of 3,800 mm². To assess flow distribution and improve velocity uniformity at the outlet, the channel was divided into 12 elemental sections (ES) (Figure S3). This division allowed for the application of convergence criteria to determine flow rates and average velocities for each section, helping to identify improvements for better velocity uniformity.

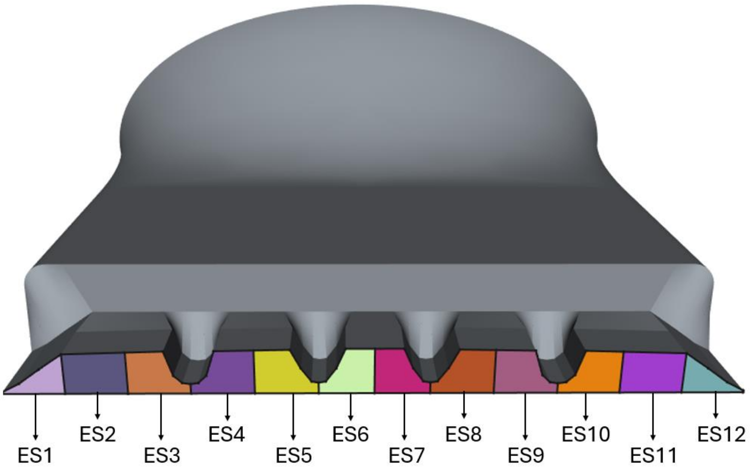

Figure S3. Division of the extrusion die flow channel outlet into Elemental Sections (ES).

 https://doi.org/10.3390/polym17111498

Similarly to previous case, the flow obstruction was designed at the pre-parallel zone of the extrusion die flow channel, as illustrated in Figure S4.

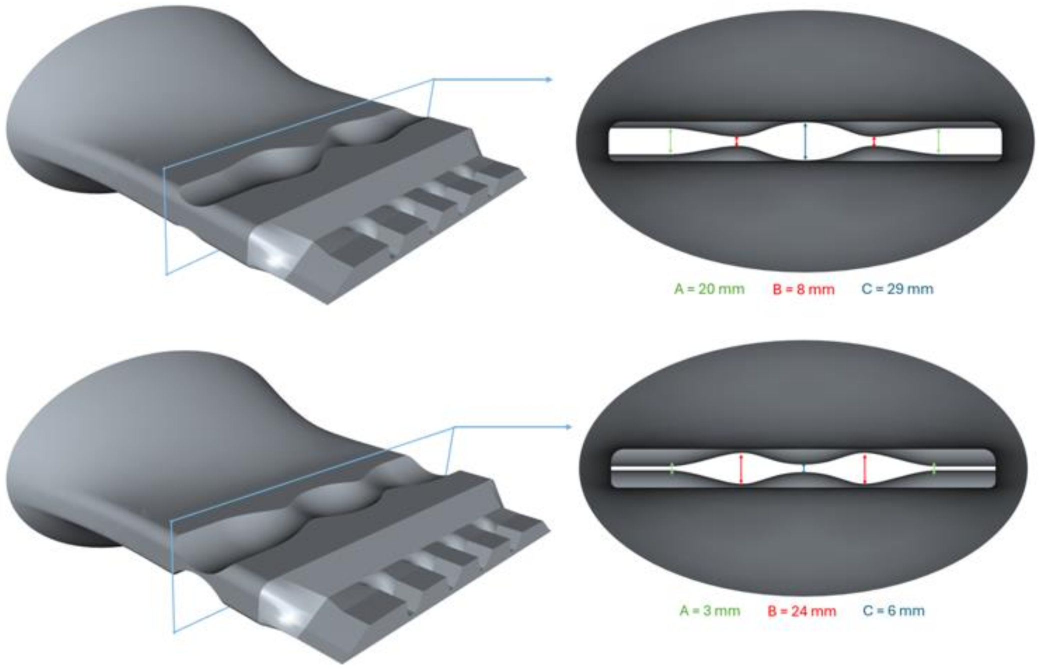

Figure S4. Location of the flow obstruction in the extrusion die flow channel, and example of different combinations of the Design Parameters and their corresponding geometry.

The flow obstruction was designed to direct the flow and control distribution at the extrusion die outlet. It was fully parameterized with three design parameters (A, B, and C), ranging from 3 mm to 30 mm, as shown in Figure S4. These parameters define the obstruction's cross-section shape, which was designed with a smooth shape defined by a spline, avoiding sharp edges and thus enhancing flow smoothness and machinability.

The material employed and imposed boundary conditions are described in Subsections 3.3 and 3.4, respectively. In this case study, the Bayesian optimization loop was defined to perform 19 iterations, thus conducting a total of 19 simulations to search for best parameters combination of A, B, and C, with the goal of minimizing the objective function defined in Section 2.4.5. The acquisition function, Expected Improvement (EI), was used to balance exploration and exploitation in the search space, with parameters ranging from 3 mm to 30 mm. The initial dataset came from a simulation with no obstructions (A = 30 mm, B = 30 mm, C = 30 mm), providing starting values for the optimization loop, as shown in Table S1, which includes reference values for pressure drop and velocity uniformity.

Table S1. Initial dataset for optimization loop for case study 2.

**A [mm]**	**B [mm]**	**C [mm]**	ΔP **[MPa]**	U_{unif}
30.00	30.00	30.00	10.3516	0.8020

Simulation results for the initial die geometry, used as dataset for the starting point for optimization, are shown in Figure S5. The velocity field (Figure S5a) indicates higher velocities at the center of the outlet and lower velocities at the lateral sections. The temperature field (Figure S5b) shows an increase in temperature towards the outlet,

particularly in high-velocity regions. The pressure field (Figure S5c) reveals a gradual decrease along the flow direction, with a total pressure drop of about 10 MPa.

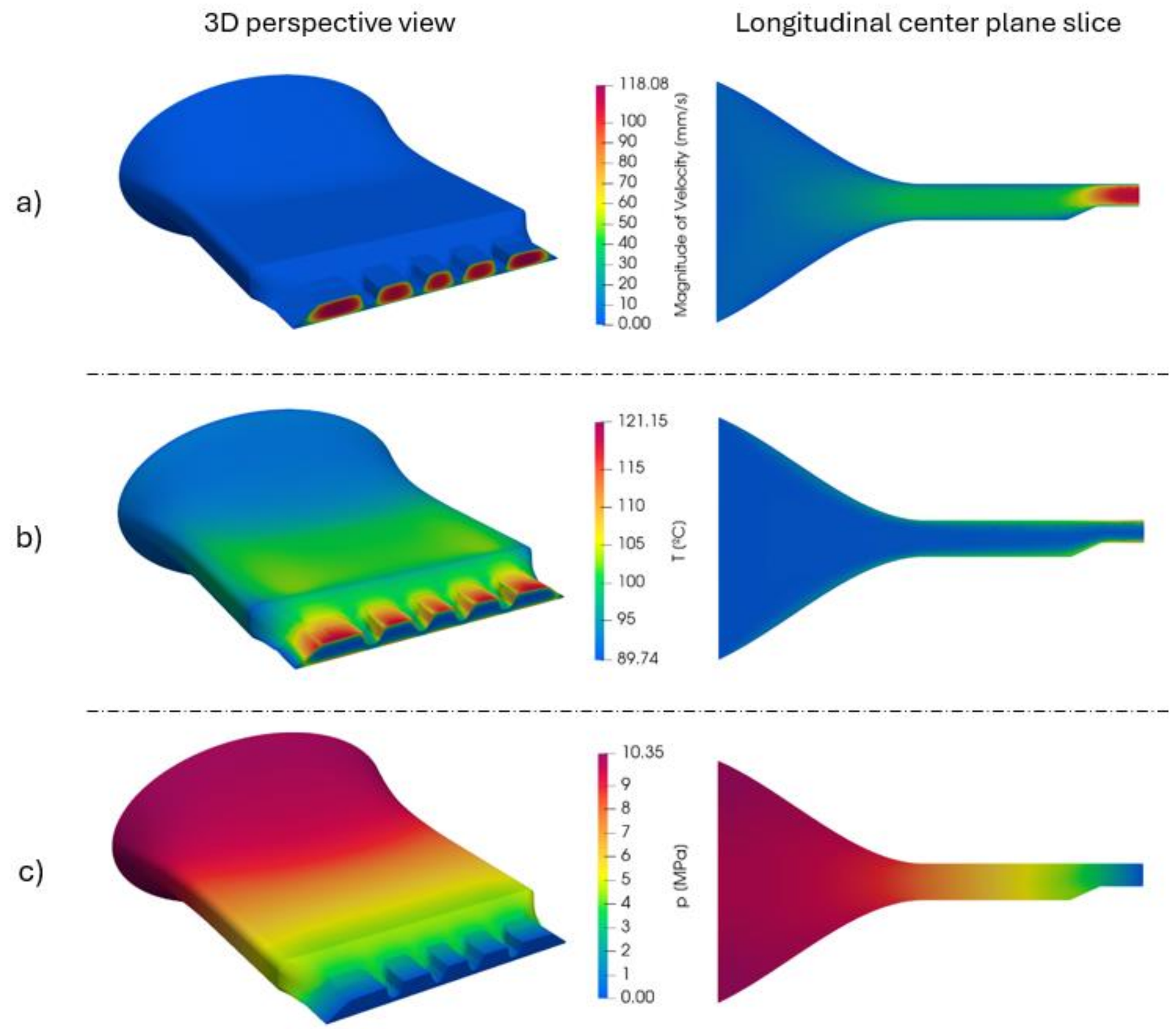

Figure S5. Simulation results of a) velocity, b) temperature, and c) pressure fields for initial dataset geometry of case study 2. Left column: full geometry 3D view; right column: longitudinal center plane slice illustrating internal flow development.

Table S2 shows the sequence of simulations conducted during the optimization process, along with the parameter combinations proposed by the Bayesian optimization algorithm and their corresponding results for pressure drop ΔP . velocity uniformity U_{unif} and objective function value F_{objOpt} .

Table S2. Optimization loop results for case study 2.

Trial	A [mm]	B [mm]	C [mm]	ΔP [MPa]	U_{unif}	F_{objOpt}
1	16.08	26.22	5.38	11.1542	0.7839	0.2214
2	26.70	13.90	16.40	11.1387	0.7586	0.2409
3	26.28	23.28	15.98	10.5381	0.7973	0.1814
4	28.10	18.04	20.60	10.6486	0.8058	0.1798

5	3.68	10.66	16.92	13.986	0.6082	0.4966
6	24.00	10.76	25.32	11.075	0.8019	0.2032
7	6.48	29.32	3.20	11.8924	0.744	0.2884
8	18.74	7.80	20.64	11.9322	0.7996	0.2456
9	24.60	20.2	17.08	10.7136	0.7959	0.1908
10	11.66	6.70	4.58	14.3409	0.7167	0.4266
11	29.20	27.0	28.76	10.1902	0.798	0.1643
12	29.34	5.80	27.00	11.0742	0.8013	0.2037
13	13.04	29.10	26.84	10.751	0.7517	0.2280
14	30.00	22.28	30.00	10.277	0.8001	0.1667
15	26.12	28.30	29.92	10.2823	0.7995	0.1675
16	22.08	6.92	24.04	10.4835	0.8002	0.2240
17	30.00	28.38	26.54	10.1525	0.7973	0.1630
18	30.00	9.52	30.00	10.7938	0.8025	0.1894
19	30.00	28.84	28.44	10.1339	0.7967	0.1626

The table shows that the optimizer explores various parameter combinations in the initial iterations, with the objective function values gradually decreasing as it converges toward optimal configurations. Figure S6 illustrates this convergence trend and the improvements in velocity uniformity over the iterations.

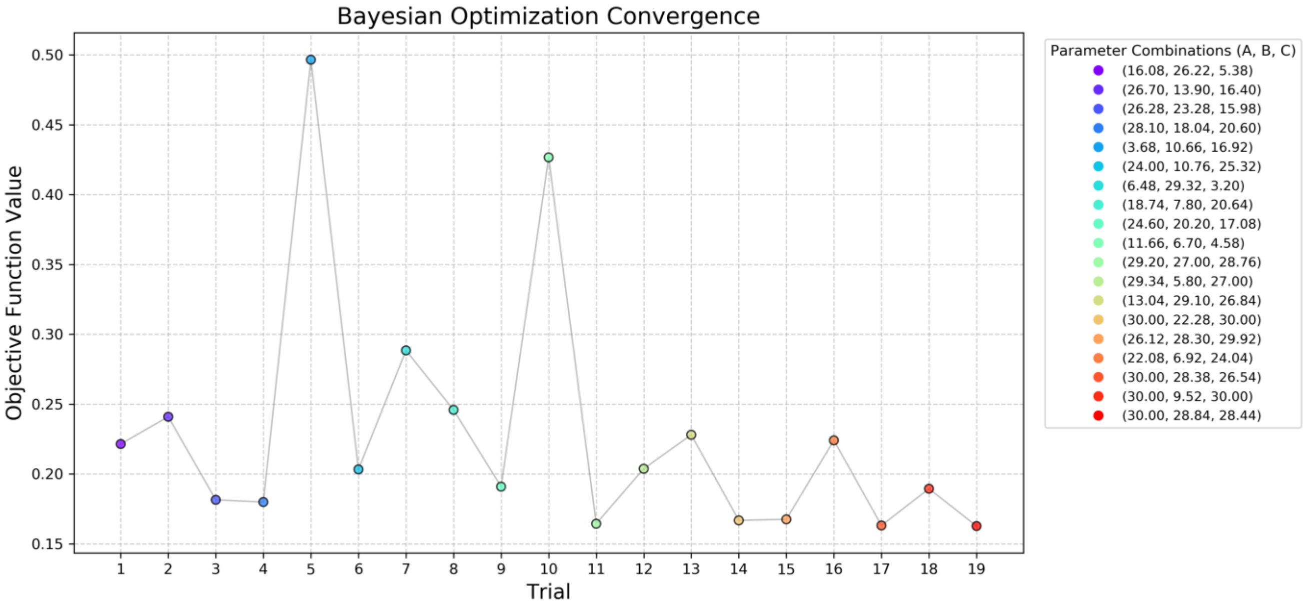

Figure S6. Objective Function evolution over the optimization trial.

In the initial iterations, the objective function values showed moderate variation, reflecting the exploration phase of the Bayesian optimizer. A peak was observed at Trial 5, with a high objective value (0.4966) due to a high pressure drop (13.986 MPa) and poor velocity uniformity (0.6082). As the optimization progressed, the search refined, focusing on promising areas, leading to a downward trend in the objective function from Trial 10 onward. The lowest objective value (0.1626) was achieved at Trial 19, indicating the most balanced configuration. Figure S7 shows the velocity distribution at the outlet across the 12 Elemental Sections (ES) for the different simulations executed within the loop.

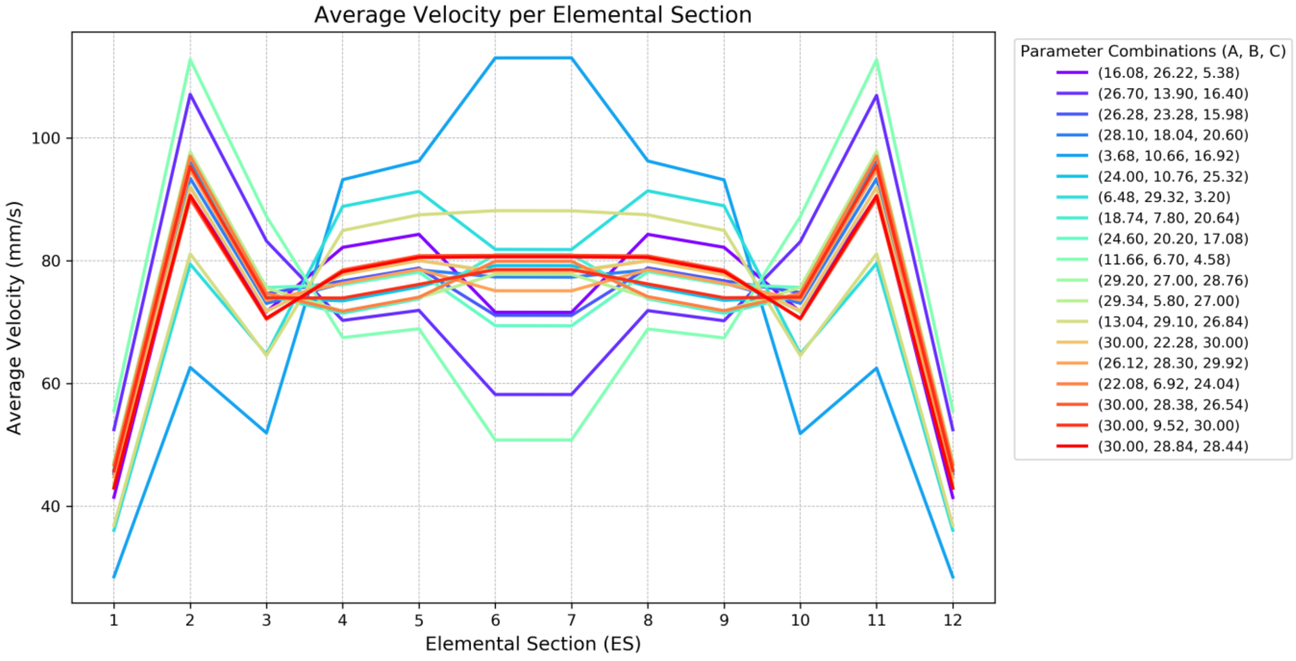

Figure S7. Elemental Sections average velocity for different parameter combinations.

Initial configurations tested showed notable velocity non-uniformity across the outlet, with the central sections often having velocities that were either too high or too low compared to the lateral sections. These uneven profiles, represented by the more erratic curves seen in the dark blue (Trial 5) and light green (Trial 10) lines, improved as the optimization continued. By the final iterations, the outlet profiles became much more uniform, with the velocity differences across the sections significantly reduced. The best performance was obtained with the geometry from Trial 19, which had values of A = 15.00 mm, B = 14.42 mm, and C = 14.22 mm. This configuration resulted in an objective function value of 0.1626, a velocity uniformity of 0.7967, and a pressure drop of 10.13 MPa.

Figure S8 illustrates the velocity magnitude distribution at the die outlet for two selected geometries: Trial 5, the worst-performing configuration, and Trial 19, the optimized geometry with the lowest objective function value. In Trial 5, the flow was highly non-uniform, with higher velocities concentrated in the central sections and stagnation in the lateral sections, leading to a noticeable imbalance. In contrast, Trial 19 displayed a much more even velocity distribution across all sections, with smoother and more balanced velocity peaks. This uniform flow pattern contributed directly to the improved performance, including better velocity uniformity and a reduced pressure drop.

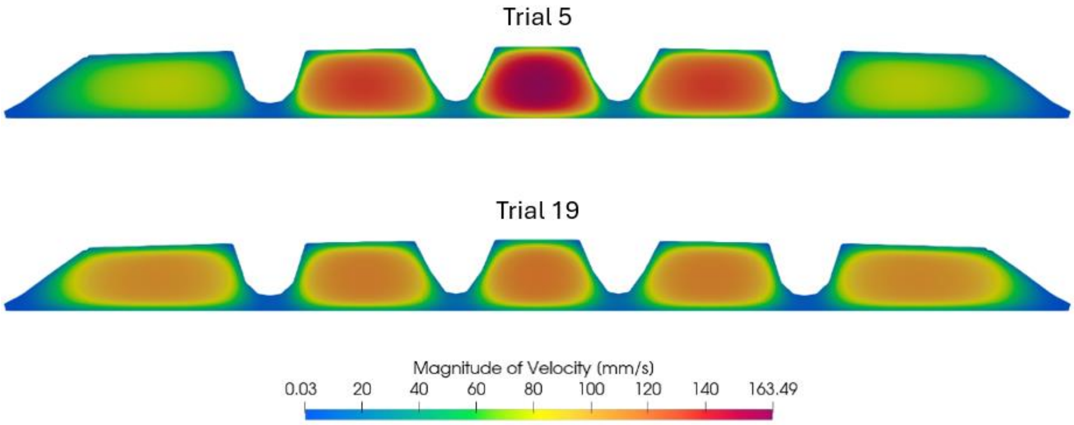

Figure S8. Die outlet velocity field comparison for Trial 5 (top) and Trial 19 (bottom) of the optimization loop, for case study 2.

Trial 19 not only achieved the best objective function value in the optimization loop but also provided better results than the baseline geometry - which lacked a flow obstruction. Although there was a slight decrease of 0.66% in outlet velocity uniformity (from 0.8020 to 0.7967), it resulted in a 2.1% reduction in pressure drop (from 10.35 MPa to 10.13 MPa). This led to an overall improvement in the objective function, from 0.1688 in the baseline geometry to 0.1626 in the optimized design, demonstrating the effectiveness of the optimization, even with multiple conflicting objectives.

If weighting factor for pressure drop was defined as 0, and thus only velocity uniformity was focused, Trial 4 would have been chosen as the optimal geometry since it provided the highest uniformity (0.8058). However, with a different objective the optimization algorithm should have explored different regions of the solution space. As for the case study performed for the complex geometry (shown in Section 3 and 4), these results demonstrate the importance of the weighting strategy in multi-objective optimization and the framework's flexibility to accommodate different design priorities. Overall, the results also demonstrate the optimizer’s ability to achieve near-ideal flow uniformity without sacrificing pressure efficiency.
