# Supplementary material for: A Novel Approach to Automatically Balance Flow in Profile Extrusion Dies Through Computational Modeling
Source: Polymers (Basel). 2025 May 28;17(11):1498. doi: 10.3390/polym17111498 (PMC12157835; doi:10.3390/polym17111498)
Supplement: Supplementary file 1 [file polymers-17-01498-s001.zip › Computational Mesh_final.pdf]

polymers
MDPI

The computational mesh, generated using the *cartesianMesh* utility from *cfMesh*, is shown in Figure S1. The meshing strategy aimed to achieve higher resolution near the die boundaries, the most critical areas for accurately capturing flow behavior due to the imposed boundary conditions. Special attention was given to the outlet region, where intense velocity gradients directly influence optimization objectives. To ensure accurate evaluation of the outlet flow distribution, this region was discretized with approximately 25 cells along the height of the tire tread profile, allowing for fine resolution of local velocity variations.

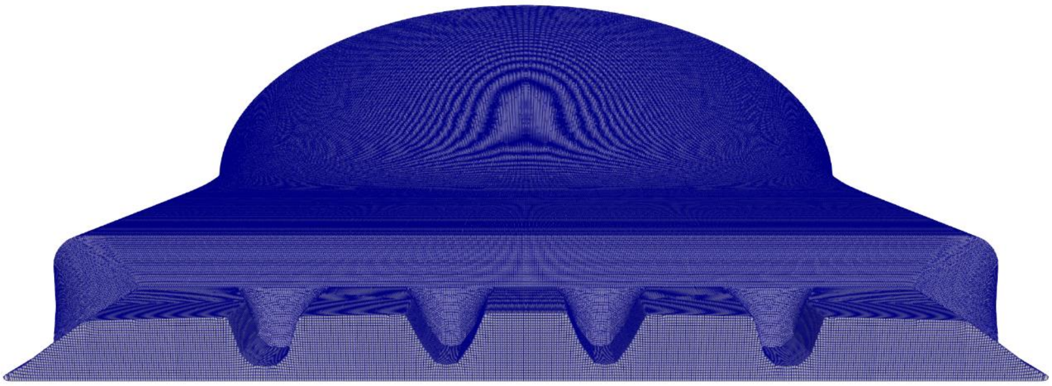

Figure S1. Extrusion die flow channel computational mesh.

To achieve this level of refinement, a mesh independence study was conducted to ensure that simulation results were not affected by the mesh details. Various mesh configurations were tested to assess their impact on pressure drop and outlet velocity uniformity. Coarser meshes in the boundary regions led to inaccurate predictions, especially near the die outlet. A comparison of two refined meshes revealed a deviation of nearly 1.5% in the objective function, confirming that the final mesh configuration was sufficiently refined for accurate and reliable results.

The final mesh comprised approximately 2.5 million cells, with higher refinement levels at the boundaries to enhance resolution where necessary while keeping computational costs reasonable.
